# Psychological distress in nursing students: relationship with screen time, diet and physical activity

**DOI:** 10.1590/1518-8345.6746.3960

**Published:** 2023-07-21

**Authors:** Enrique Ramón-Arbués, José Manuel Granada-López, Pedro José Satústegui-Dordá, Emmanuel Echániz-Serrano, Lucía Sagarra-Romero, Isabel Antón-Solanas

**Affiliations:** 1 Universidad San Jorge, Facultad de Ciencias de la Salud, Villanueva de Gállego, Aragón, España.; 2 Universidad de Zaragoza, Facultad de Ciencias de la Salud, Zaragoza, Aragón, España.

**Keywords:** Nursing Students, Anxiety, Depression, Motor Activity, Diet, Cross-Sectional Studies, Estudiantes de Enfermería, Ansiedad, Depresión, Actividad Motora, Dieta, Estudios Transversales, Estudantes de Enfermagem, Ansiedade, Depressão, Atividade Motora, Dieta, Estudos Transversais

## Abstract

**Objective:**

to determine the prevalence of anxiety and depression symptoms, as well as their association with screen time, diet and physical activity, in a cohort comprised by nursing students.

**Method:**

a descriptive and cross-sectional study conducted with a sample of 648 Nursing students. The instruments used were the Hospital Anxiety and Depression Scale, the short version of the International Physical Activity Questionnaire and the Spanish Healthy Eating Index (Índice *de Alimentación Saludable Español*, IASE). Different multiple linear regression models were performed to analyze the association between anxiety/depression symptoms and screen time, diet and physical activity.

**Results:**

the prevalence values for anxiety and depression symptoms were 24.1% and 3.7%, respectively. Prolonged screen times (>6 hours a day), low levels of physical activity and inadequate diet were independently and significantly associated with deeper psychological symptoms.

**Conclusion:**

Nursing students’ mental health might benefit from the implementation of initiatives promoting healthy lifestyles.

## Abstract (es)

**Objetivo:**

determinar la prevalencia de síntomas de ansiedad y depresión, al igual que su asociación con el tiempo de pantalla, la dieta y la actividad física, en una cohorte de estudiantes de enfermería.

**Método:**

estudio descriptivo y transversal sobre una muestra de 648 estudiantes de enfermería. Se utilizaron la *Hospital Anxiety and Depression Scale*, la versión corta del *International Physical Activity Questionnaire* y el Índice de Alimentación Saludable español. Se llevaron a cabo diferentes modelos de regresión lineal múltiple para analizar la asociación entre los síntomas de ansiedad y depresión, y el tiempo de pantalla, la dieta y la actividad física.

**Resultados:**

la prevalencia de síntomas de ansiedad y depresión fue de 24,1% y 3,7%, respectivamente. El tiempo de pantalla prolongado (>6 horas por día), baja actividad física y dieta inadecuada se asociaron de manera independiente y significativa con una mayor sintomatología psicológica.

**Conclusión:**

la salud mental de los estudiantes de enfermería podría beneficiarse de implementarse iniciativas que promuevan estilos de vida saludables.

## Abstract (pt)

**Objetivo:**

determinar a prevalência de sintomas de ansiedade e depressão e sua associação com tempo de tela, dieta e atividade física em uma coorte de estudantes de enfermagem.

**Método:**

estudo descritivo transversal com amostra de 648 estudantes de enfermagem. Utilizou-se a *Hospital Anxiety and Depression Scale*, a versão curta do *International Physical Activity Questionnaire* e o Índice Espanhol de Alimentação Saudável. Foram realizados diferentes modelos de regressão linear múltipla para analisar a associação entre sintomas de ansiedade e depressão e tempo de tela, dieta e atividade física.

**Resultados:**

a prevalência de sintomas de ansiedade e depressão foi de 24,1% e 3,7%, respectivamente. O tempo prolongado de tela (>6 horas por dia), a baixa atividade física e a dieta inadequada foram associados de forma independente e significativa ao aumento da sintomatologia psicológica.

**Conclusão:**

a saúde mental dos estudantes de enfermagem pode ser beneficiada com a implementação de iniciativas que promovam estilos de vida saudáveis.

**Highlights:**

**(1)** Anxiety and depression signs are usual in nursing students.

**(2)** Deficient diet, sedentary lifestyle and low levels of physical activity are prevalent behaviors.

**(3)** Diet, screen time and physical activity are related to psychological distress.

**(4)** It is necessary to monitor and promote future nurses’ mental health.

## Introduction

Mental health problems are one of the leading causes of disability and an increasing public health issue at the global level due to disease progressions, their increased prevalence and the difficulties associated with their therapeutic management^(^ 1 ^)^. On many occasions, mental health problems appear in early adulthood and, in this life phase, they are associated with higher incidence of medium- and long-term physical and emotional problems^(^ 2 ^)^, with difficulties entering the labor market^(^ 3 ^)^, with worse sleep quality^(^ 4 ^)^ or with dysfunctional social relationships^(^ 5 ^)^, among others.

University students are faced with important challenges, risks and transitions inherent to social development that increase the risk of suffering mental health problems^(^ 6 ^)^. In addition, the stressors can be even greater in the case of future nurses. Clinical practices imply close human contact based on deep emotional commitment, and may include dealing with severe diseases and deaths^(^ 7 ^)^.

Previous studies have characterized psychological distress by assessing depression and anxiety in nursing students at the global level, reporting high results, although significantly dissimilar^(^ 8 ^-^ 10 ^)^. From this perspective, it is of special importance to monitor and early detect psychological distress in nursing students, as well as its associated factors. In this sense, both in the clinical population and among adolescents and young individuals, several research studies have observed certain associations between specific behaviors and anxiety/stress. Among them, inadequate diet^(^ 11 ^-^ 12 ^)^ and sedentary lifestyle with considerable time devoted to using devices with a screen^(^ 13 ^-^ 14 ^)^ seem to be associated with greater psychological distress. On the other hand, physical activity seems to exert a protective mechanism against anxiety and depression. However, these relationships are yet to be confirmed in specific populations such as university students and, more in particular, nursing students.

Based on these deficits, the objective of this study was to characterize psychological distress in local nursing students, based on determining the prevalence of anxiety and depression symptoms, as well as their association with screen time, physical activity and quality of the diet.

## Method

### Study design and locus

A descriptive and cross-sectional study was conducted with a sample comprised by nursing students enrolled in any of the two institutions that offer this degree in the Aragon Region (Spain), namely: *Universidad de Zaragoza* and *Universidad San Jorge*.

### Population

Calculation of the minimum sample size required to meet our main objective of determining the prevalence of anxiety and depression was based on results recently obtained through the Hospital Anxiety and Depression Scale (HADS) in a sample of Nursing students from the Principality of Asturias (Northern Spain)^(^ 15 ^)^. Consequently, for a 95% confidence level and 2% absolute precision, a required minimum of 610 participants was estimated for our study.

Recruitment of the participants and data collection were conducted in the classrooms from September to November 2022. Thus, the students were informed about the study nature and objectives, the voluntary character of their participation and about how the data obtained would be managed based on the anonymity criterion. From an eligible enrolled population of 1,182 students, 682 consented to take part in this study and answered the required questionnaires (response rate: 57.6%). Of all 682 questionnaires received, 34 were considered as not valid (generalized absence of data or overtly unreal data) and were consequently excluded from the analysis (rejection rate: 5.0%).

### Variables and instruments used for data collection

The data collection questionnaire had three sections: 1. Sociodemographic and academic data; 2. Lifestyles and 3. Psychological symptomatology (anxiety and depression). In the first section, which was prepared *ad hoc*, the participants reported information about their age, gender, housing arrangement (living alone, living with roommates/partner, living with parents/family members), work situation (unemployed, part-time job, full-time job), perceived economic level (low/very low, average, high/very high), academic year, and mean grade throughout the course.

Screen time, quality of the diet and physical activity were assessed in the second section. Screen time was evaluated using the following question: “*On a normal day, how much time do you devote to using devices with a screen (smartphone, tablet, computer, television, etc.?*”. Based on the answers to this question, the participants were categorized into 3 groups by use time (<3 hours/day, 3-6 hours/day, >6 hours/day).

Physical activity was evaluated by means of the International Physical Activity Questionnaire-Short Form (IPAQ-SF). IPAQ-SF provides information about the intensity, frequency and duration of the physical activity performed in the last 7 days. This questionnaire offers both quantitative and qualitative information about the physical activity performed. In the quantitative analysis, the unit of measure was Metabolic Equivalent of Task (MET) *per* week, where 1 MET is the energy expenditure derived from the at-rest metabolic level. In the qualitative study, the official interpretation protocol of the tool^(^ 16 ^)^ allows classifying the population into 3 activity levels (low, moderate, high). IPAQ-SF has been validated for the Spanish university population, showing a satisfactory correlation (0.69) with the accelerometer^(^ 17 ^)^.

The participants’ eating habits were assessed with the Spanish Healthy Eating Index (Índice *de Alimentación Saludable Español*, IASE)^(^ 18 ^)^. This tool is an adaptation of the Healthy Eating Index^(^ 19 ^)^ to the Spanish context according to the recommendations proposed by the Spanish Society of Community Nutrition. The index has 10 items (scoring range: 0-10) for a final score varying from 0 to 100 points. Based on the score obtained, the participants are categorized as follows: scores >80 points (healthy diet), between 50 and 80 (diet requiring changes), and scores <50 (inadequate diet). This questionnaire is repeatedly used in Spanish university populations^(^ 20 ^-^ 21 ^)^. Although it has not yet been validated for the Spanish university population, the original Healthy Eating Index questionnaire has indeed been validated through plasma biomarkers, showing satisfactory correlation levels ranging from a minimum of r=0.23 (for cereals) to a maximum of r=0.71 for food variety^(^ 22 ^)^.

Finally, the anxiety and depression symptoms were assessed with the HADS scale^(^ 23 ^)^, a tool originally conceived for the clinical population, although it is currently widely employed and has proved to be useful in the community scope. HADS consists of two 6-item subscales: HADS-Depression and HADS-Anxiety. Each item is classified following a 4-point Likert (from 0 = Never to 3 = Almost all the time), for a global score in each subscale ranging from 0 to 21. Based on this result, possible depression and possible anxiety were considered for the participants with scores ≥8, whereas probable depression and probable anxiety for those with values ≥11 in the respective subscales^(^ 24 ^)^. In its validation study on the Spanish population, this questionnaire has shown optimum properties for anxiety and depression screening^(^ 25 ^-^ 26 ^)^.

### Data analysis

The descriptive analysis corresponding to the characteristics of the sample (sociodemographic, academic, lifestyle-related, and psychological symptomatology) is presented through mean values and standard deviations for the quantitative variables, and with numbers and percentages for the categorical ones. The bivariate comparison of those characteristics based on gender was performed through the Student’s t test for the quantitative variables using the χ^2^ test (or Fisher’s Exact test when applicable) for the categorical ones.

In addition, several multiple linear regression models were performed with the objective of determining the association degree between screen time, physical activity, diet and anxiety/depression symptoms, both for the total population and by sex. The models performed on the total sample were adjusted by age, sex, economic situation, mean grade, academic year, housing arrangement and work situation, as well as by screen time and diet (for physical activity) or by physical activity and diet (for screen time) or by physical activity and screen time (for diet). The models disaggregated by sex were adjusted by age, economic situation, mean grade, academic year, housing arrangement and work situation, as well as by screen time and diet (for physical activity) or by physical activity and diet (for screen time) or by physical activity and screen time (for diet). All calculations were made in the Statistical Package for the Social Sciences (SPSS), Version 23.0, accepting p-values<0.05 as statistically significant.

### Ethical considerations

Prior to initiating the study, due authorization was obtained from the Aragon Clinical Research Ethics Committee. Initially, the participants were informed about the objectives, methodology and potential risks arising from their participation in the study and about the possibility of withdrawing from the research at any moment. Subsequently, and prior to data collection, all the participants signed the informed consent form.

## Results

### Characteristics of the sample

A total of 648 nursing students comprised the final study sample. The participants’ mean age was 21.7 years old and the female gender prevailed (84.2%). Most of the participants were enrolled in 2^nd^ or 3^rd^ year of the course (50.3%), lived with their parents/family members (60.2%), perceived their economic level as average (85,2%) and had no paid jobs (76.9%). By sex, no statistically significant differences were observed in relation to age, academic year and grade or work situation. However, men were more prone to living alone than women (p<0.05) (Table 1).

**Table 1 -:** Characteristics of the participants (n=648). Zaragoza, AR, Spain, 2022

Variable	Total (n=648)	Women (n=546)	Men (n=102)	P
Age (mean±standard deviation)	21.7±4.7	21.6±4.7	22.1±4.3	0.345
Housing arrangement				
Lives alone	48 (7.4%)	30 (5.5%)	18 (17.6%)	0.001
Lives with roommates/partner	210 (32.4%)	180 (33.0%)	30 (29.4%)	
Lives with parents/family members	390 (60.2%)	336 (61.5%)	54 (52.9%)	
Academic year				
1^st^ year	163 (25.1%)	131 (24.0%)	32 (31.3%)	0.288
Intermediate years	326 (50.3%)	279 (51.1%)	47 (46.0%)	
Last year	159 (24.5%)	136 (24.9%)	23 (22.5%)	
Mean grade (mean±standard deviation)	7.1±0.6	7.1±0.6	7.0±0.5	0.085
Perceived socioeconomic level				
Very low/Low	66 (10.2%)	60 (11.0%)	6 (5.9%)	0.011
Average	552 (85.2%)	456 (83.5%)	96 (94.1%)	
High/Very high	30 (4.6%)	30 (5.5%)	0 (0.0%)	
Work situation				
No paid job	498 (76.9%)	420 (76.9%)	78 (76.5%)	0.794
Full-time job	30 (4.6%)	24 (4.4%)	6 (5.9%)	
Part-time job	120 (18.5%)	102 (18.7%)	18 (17.6%)	

### Physical activity, screen time and diet

Assessed by means of IASE, the quality of the participants’ diet turned out to be deficient. Up to 93.5% reported inadequate diet or requiring changes. The time spent in front of screens was high (5.8 hours a day on average), with a vast majority of the participants (72.2%) indicating daily times of more than 3 hours. 51.9% of the participants reported low physical activity, whereas only 23.1% reported high levels. By sex, the men presented not-so-negative diets, with higher IASE scores and more chances of following a healthy diet. They also showed lower use of devices with a screen and greater willingness for physical activity, as evidenced by their higher weekly metabolic expenditure and by the higher proportion of men with moderate and high levels of physical activity (p<0.05) (Table 2).

**Table 2 -:** Physical activity, screen time and diet among the participants (n=648). Zaragoza, AR, Spain, 2022

Variable	Total (n=648)	Women (n=546)	Men (n=102)	p
Diet				
Healthy Eating Index (mean±standard deviation)	54.7±14.7	53.6±13.9	60.8±17.1	0.000
Healthy diet	42 (6.5%)	30 (5.5%)	12 (11.8%)	0.000
Diet requiring changes	342 (52.8%)	276 (50.5%)	66 (64.7%)	
Inadequate diet	264 (40.7%)	240 (44.0%)	24 (23.5%)	
Screen time				
Hours a day (mean±standard deviation)	5.8±1.7	5.9±1.8	5.4±1.5	0.007
<3 hours/day	180 (27.8%)	138 (25.3%)	42 (41.2%)	0.001
3-6 hours/day	378 (58.3%)	324 (59.3%)	54 (52.9%)	
>6 hours/day	90 (13.9%)	84 (15.4%)	6 (5.9%)	
Physical activity				
METs^*^-week (mean±standard deviation)	2,178.7±1,802.3	2,040.2±1,626.6	2,920.1±2,423.4	0.001
Low level of physical activity	336 (51.9%)	300 (54.9%)	36 (35.3%)	0.001
Moderate level of physical activity	162 (25.0%)	132 (24.2%)	30 (29.4%)	
High level of physical activity	150 (23.1%)	114 (20.9%)	36 (35.3%)	

^*^MET = Metabolic Equivalent of Task

### Anxiety and depressive symptoms

The mean scores obtained in the HADS-A and HADS-D scales were 8.34±3.30 and 4.12±2.82, respectively. Only 47.2% of the participants did not present any type of symptoms compatible with anxiety, with confirmation of the anxious symptomatology in up to 24.1% of the participants. Referring to depression, 88% of the participants did not show depressive symptoms, and only 3.7% had clinical signs compatible with depression (Table 3). By sex, the scores obtained in HADS indicated greater anxiety and depression traits in women and men, respectively (p<0.05), although the probability of finding severe clinical signs of depression was slightly higher in the women’s group.

**Table 3 -:** Anxiety and depression symptoms in the sample (n=648). Zaragoza, AR, Spain, 2022

Variable	Total (n=648)	Women (n=546)	Men (n=102)	p
HADS-A^*^ score (mean±standard deviation)	8.34±3.30	8.39±3.39	8.05±2.74	0.010
No anxiety	306 (47.2%)	258 (47.3%)	48 (47.1%)	0.000
Possible anxiety	186 (28.7%)	138 (25.3%)	48 (47.1%)	
Anxiety	156 (24.1%)	150 (27.5%)	6 (5.9%)	
HADS-D^†^ score (mean±standard deviation)	4.12±2.82	4.07±2.91	4.41±2.26	0.106
No depression	570 (88.0%)	486 (89.0%)	84 (82.4%)	0.018
Possible depression	54 (8.3%)	36 (6.6%)	15 (14.7%)	
Depression	24 (3.7%)	24 (4.4%)	3 (2.9%)	

^*^HADS-A = Hospital Anxiety and Depression Scale-Anxiety; ^†^HADS-D = Hospital Anxiety and Depression Scale-Depression

### Association between physical activity, screen time, diet and psychological symptoms

All three multivariate analysis models (for the total and by gender) performed to evaluate the association between physical activity, screen time, diet and the anxiety symptoms showed that low levels of physical activity, prolonged screen times (>6 hours) and unhealthy diets (diet requiring changes and inadequate diet) are statistically and significantly associated with higher scores in the HADS-A anxiety scale. These behaviors are maintained both for the general sample and in the analysis disaggregated by gender. The predictive capacity of these three models ranged between 19.5% and 67.4% (Table 4).

**Table 4 -:** Multiple linear regression of factors associated with the scores obtained in the HADS-A^*^ scale (n=648). Zaragoza, AR, Spain, 2022

Variable	Total^†^	Women^‡^	Men^‡^
	B coefficient (95% Confidence Interval)	B coefficient (95% Confidence Interval)	B coefficient (95% Confidence Interval)
Physical activity (Reference group: Moderate)			
Low level of physical activity	0.633 (0.039; 1.227)^§^	0.451 (-0.192; 1.094)	2.359 (0.543; 4.175)^§^
High level of physical activity	-0.955 (-1.886; -0.097)^§^	-0.452 (-1.246; 0.343)	-3.142 (-4.268; -2.016)^§^
Screen time (Reference group: 3-6 hours/day)			
<3 hours/day	0.083 (-0.651; 0.818)	0.398 (-0.393; 1.189)	-3.391 (-4.401; -2.381)^§^
>6 hours/day	1.507 (0.701; 2.311)^§^	1.232 (0.622; 1.841)^§^	2.705 (1.044; 4.366)^§^
Diet (Reference group: Healthy diet)			
Diet requiring changes	1.409 (0.415; 2.404)^§^	0.895 (-0.292; 2.082)	3.911 (2.565; 5.256)^§^
Inadequate diet	1.919 (0.886; 2.953)^§^	1.342 (0.138; 2.546)^§^	8.582 (6.774; 10.390)^§^
R^2^ coefficient (Corrected R^2^)	0.209 (0.195)	0.244 (0.229)	0.710 (0.674)

^*^HADS-A = Hospital Anxiety and Depression Scale-Anxiety; ^†^Model adjusted by age, gender, economic situation, mean grade, academic year, housing arrangement and work situation, as well as by screen time and diet (for physical activity) or by physical activity and diet (for screen time) or by physical activity and screen time (for diet); ^‡^Model adjusted by age, economic situation, mean grade, academic year, housing arrangement and work situation, as well as by screen time and diet (for physical activity) or by physical activity and diet (for screen time) or by physical activity and screen time (for diet); ^§^p<0.05

Referring to the association between physical activity, screen time, diet and depressive symptoms, the different multiple linear regression models developed evidenced that longer screen times (>6 hours) and diets not following the recommendations (diet requiring changes and inadequate diet) are statistically and significantly associated with higher scores in the HADS-D scale. The behavior of these associations was similar in men and in women. However, an inverse association between physical activity and depression was only observed in the men’s group. Thus, the higher the physical activity level, the fewer the depressive symptoms (lower HADS-D scores) in men. The predictive capacity of these models ranged between 20% and 78% (Table 5).

**Table 5 -:** Multiple linear regression of factors associated with the scores obtained in the HADS-D^*^ scale (n=648). Zaragoza, AR, Spain, 2022

Variable	Total^†^	Women^‡^	Men^‡^
	B coefficient (95% Confidence Interval)	B coefficient (95% Confidence Interval)	B coefficient (95% Confidence Interval)
Physical activity (Reference group: Moderate)			
Low level of physical activity	0.369 (-0.307; 1.033)	0.551 (-0.010; 1.113)	-0.437 (-1.668; 0.793)
High level of physical activity	-0.658 (-1.210; 0.131)	0.004 (-0.690; 0.697)	-3.101 (-3.864; -2.338)^§^
Screen time (Reference group: 3-6 hours/day)			
<3 hours/day	-0.116 (-0.759; 0.527)	0.053 (-0.479; 0.586)	-0.854 (-1.979; 0.271)
>6 hours/day	0.862 (0.172; 1.552)^§^	0.696 (0.005; 1.387)^§^	1.588 (0.904; 2.273)^§^
Diet (Reference group: Healthy diet)			
Diet requiring changes	1.553 (0.716; 2.390)^§^	1.918 (0.882; 2.955)^§^	0.020 (-0.891; 0.932)
Inadequate diet	1.653 (0.784; 2.523)^§^	1.869 (0.818; 2.921)^§^	1.419 (0.194; 2.644)^§^
R^2^ coefficient (Corrected R^2^)	0.231 (0.217)	0.215 (0.199)	0.803 (0.779)

^*^HADS-D = Hospital Anxiety and Depression Scale-Depression; ^†^Model adjusted by age, gender, economic situation, mean grade, academic year, housing arrangement and work situation, as well as by screen time and diet (for physical activity) or by physical activity and diet (for screen time) or by physical activity and screen time (for diet); ^‡^Model adjusted by age, economic situation, mean grade, academic year, housing arrangement and work situation, as well as by screen time and diet (for physical activity) or by physical activity and diet (for screen time) or by physical activity and screen time (for diet); ^§^p<0.05

## Discussion

The objective of this research was to examine the prevalence of anxiety and depression in a sample comprised by Spanish nursing students, as well as their association with screen time, physical activity and quality of the diet. We were able to detect high prevalence of risk behaviors for health, such as low levels of physical activity (51.9%), deficient diet (40.7%) and prolonged screen times (13.9%) among local future nurses. These values are similar to those already obtained in samples comprised by Spanish university students^(^ 27 ^-^ 28 ^)^. They are high prevalence values for unhealthy lifestyles, which deserves double consideration in the case of nursing students. On the one hand, unhealthy habits predict medium- and long-term health problems for these young people. On the other hand, the nursing staff is a reference in relation to health education and the promotion of healthy lifestyles. In this sense, the already published literature suggests that the health professionals’ behaviors exert an influence on their health promotion practices^(^ 29 ^)^. It is worth noting that these behaviors were associated with psychological distress. Thus, high levels of physical activity were inversely associated with presence of anxiety and depression symptoms. This relationship has already been reported in various populations^(^ 30 ^)^ and may be sustained from a neurobiological point of view due to the activating effect of physical activity on the endocannabinoid system and the brain-derived neurotrophic factor^(^ 31 ^)^.

Previous research studies have evidenced significant associations between healthy eating habits (moderate calorie intake, having breakfast or not eating snacks, among others) or intake of certain food products and nutrients (vegetables and fruit, polyunsaturated fatty acids, certain minerals or vitamins, among others) and good psychological health^(^ 32 ^-^ 33 ^)^. In this same line, the quality of our participants’ diet was inversely and strongly associated with presence of anxiety and depression.

Finally, prolonged screen times (>6 hours a day) were associated with worse mental health results in our sample. Screen time is a variable of interest nowadays, as it contributes two types of information. On the one hand, data related to use/abuse of new technologies and, on the other hand, an indirect measure of the presence of a sedentary life pattern. In this sense, excessive use of new screen-based technologies (social networks, smartphones, etc.) and their associated sedentary lifestyle have already been related to psychological distress, especially in the young population^(^ 34 ^-^ 35 ^)^.

A recent meta-analysis published in 2022 and conducted with an aggregated sample of more than 100,000 subjects determined 33.6% and 39.0% prevalence of depression and anxiety symptoms in university students, respectively^(^ 36 ^)^. Comparing these values with those obtained in cross-sectional studies, such as in this case, is a difficult task given the heterogeneity of socioeconomic contexts and the variety of diagnostic instruments used. In any case, they are higher numbers than those observed in our sample of nursing students, where approximately one-fourth of the participants presented some type of psychological distress, with higher prevalence of anxiety (24.1%) than of depression (3.7%) symptoms. These results seem to be contrary to the theory which asserts that students from the health area present higher psychological distress levels than others for being subjected to more stressors, including contact with diseases and deaths^(^ 37 ^)^.

Several studies have already analyzed the prevalence of psychological distress in nursing students, with very heterogeneous results according to the participants’ countries of origin. For example, in Canada, depression and anxiety symptoms have been observed in 32% and 39% of the students, respectively^(^ 38 ^)^. These values differ from those obtained in Turkey (depression symptoms in 55.5% and anxiety symptoms in 50.9%)^(^ 39 ^)^, Brazil (depression symptoms in 54.2% and anxiety symptoms in 40.1%)^(^ 40 ^)^, Japan (depression symptoms in 18.3% and anxiety symptoms in 34.6%)^(^ 41 ^)^ or Saudi Arabia (depression symptoms in 43.3% and anxiety symptoms in 37.2%)^(^ 42 ^)^, among others. In general terms, they are higher numbers than those obtained in this study. A possible explanation for the lower prevalence of psychological distress in our sample can be the time when the data were collected. Whereas the bulk of the recent bibliography on psychological distress in nursing students is circumscribed to the confinement period and to the effects of the COVID-19 pandemic, this study determines the psychological symptoms at a post-pandemic euphoric moment after the removal of all restrictions in Spain.

This research study has some limitations that are worth noting. In the first place, the sample was fully extracted from a single locus, the Aragon Region, which can raise doubts when extrapolating the results to the global population of Spanish nursing students. However, the participants’ gender and age profiles in this study coincide with those existing in the bulk of the Spanish nursing training programs. Secondly, our cross-sectional design only allows establishing associations, precluding causality relationships. Future research studies with longitudinal designs might provide better grounds to understand the associations observed in this survey, especially regarding how the health-related behaviors of nursing students can affect their psychological health, and vice versa. Despite these limitations, several factors lead us to believe that our results may be useful and serve as the starting point for future initiatives that promote mental health in the university context. The use of standardized data collection procedures and of validated questionnaires both to assess the psychological symptoms and to determine health-related behaviors, as well as the plausibility of the associations established, support this assumption.

To the best of our knowledge, this is the first study that analyzes depression and anxiety symptoms, as well as their association with various health-related behaviors, in a broad sample of Spanish nursing students. The results obtained in this study report considerable prevalence values for psychological distress and unhealthy behaviors among these students, which demand attention both from those responsible for formulating health policies and from the university authorities. From this perspective, it is imperative to implement new mental health monitoring and promotion activities and healthy behaviors in the university setting. Currently, students can usually access certain support services, such as tutoring or counseling. However, they are not specific to any given academic discipline or based on scientific evidence. In this sense, some authors suggest certain strategies capable of minimizing psychological distress, such as behavioral interventions^(^ 43 ^)^, mindfulness^(^ 44 ^)^ or mentoring programs^(^ 45 ^)^. In addition, nursing students subjected to stressful situations in their care practice internships might be benefited if simulation scenarios were incorporated into the curriculum. These spaces may help nursing students to gain confidence, anticipate situations and develop effective coping strategies for their future performance in emotionally complex situations^(^ 46 ^)^.

## Conclusion

The results of this study show considerable prevalence of depression and anxiety symptoms in Spanish nursing students. In addition, these symptoms are associated with low levels of physical activity, prolonged screen times and unhealthy diets. These data suggest two important problems towards the future. The first one refers to the future nurses’ medium- and long-term health. The second one concerns their future performance as nursing professionals, which can be impaired by lower promotion of their patients’ health (a person cannot so vehemently promote something they do not comply with) and by greater exposure to the burnout phenomenon, which is more incident in professionals with a less favorable baseline psychological status. These predictions denote the importance of implementing strategies that promote general and mental health in the university setting.
